# Discoid Lupus Erythematosus Lesions Associated with Systemic Fluorouracil Agents: A Case Report and Review

**DOI:** 10.7759/cureus.7828

**Published:** 2020-04-25

**Authors:** Philip R Cohen

**Affiliations:** 1 Dermatology, San Diego Family Dermatology, National City, USA

**Keywords:** capecitabine, cutaneous, discoid, erythematosus, fluorouracil, lupus, systemic, subacute, tegafur, uracil

## Abstract

Systemic fluorouracil agents include not only 5-fluorouracil (5FU), but also capecitabine, tegafur, and uracil/tegafur (UFT). Systemic lupus erythematosus (SLE), subacute cutaneous lupus erythematosus (SCLE), and discoid lupus erythematosus (DLE) are subtypes of lupus erythematosus; drug-induced lupus erythematosus can also present in each of these subtypes. This report describes the case of a 65-year-old woman with systemic 5FU-induced DLE. Fluorouracil agent-induced DLE lesions occurring after initiating treatment with either systemic 5FU or its prodrugs have been described in 19 individuals (Including the woman in this report) in the literature: tegafur (10 patients), UFT (six patients), systemic 5FU (two patients), and capecitabine (one patient). The mean duration before the appearance of the DLE lesions on sun-exposed areas was 232 days after beginning the fluorouracil agent; however, the much earlier (three weeks) appearance of the DLE lesions after starting systemic 5FU in the women described in this report may have occurred since there was no delay associated with the conversion of a precursor drug to 5FU. Within two months (mean: 36 days) after stopping the fluorouracil agent, the DLE lesions resolved in 95% of the patients. Laboratory studies were only performed on some of the patients. None of the patients tested had antibodies to Ro/Sjogren’s syndrome A (Ro/SSA) and La/Sjogren’s syndrome B (La/SSB). The antinuclear antibody (ANA) titer was elevated in 71% of the tested individuals and decreased in all of the patients who were evaluated after the causative drug was discontinued. The pathogenesis for fluorouracil agent drug-induced DLE remains to be definitively established.

## Introduction

5-fluorouracil (5FU) is a fluorinated pyrimidine. Its precursor drugs include capecitabine and tegafur. Uracil/tegafur (UFT) is a combination drug consisting of uracil and tegafur; the degradation of 5FU by dihydropyrimidine dehydrogenase is slowed by uracil. Systemic 5FU has been used to treat cancer of the breast, head and neck, gastrointestinal tract (including stomach, colon, and rectum), and pancreas [1].

Lupus erythematosus is an autoimmune disease. There are three subtypes of lupus erythematosus: systemic lupus erythematosus (SLE), subacute cutaneous lupus erythematosus (SCLE), and discoid lupus erythematosus (DLE), which is also referred to as chronic cutaneous lupus erythematosus. These same subtypes are also associated with lupus erythematosus induced by drugs [2,3].

Several cutaneous adverse events, including drug-induced lupus, are associated with 5FU [4]. This report describes the case of a 65-year-old woman with breast cancer who received systemic 5FU and developed drug-induced DLE. In addition, the report also reviews features of DLE associated not only with 5FU but also its related agents.

## Case presentation

A 65-year-old Caucasian woman presented for evaluation of an asymptomatic facial rash. Her past medical history was significant for multiple basal cell carcinomas, chondromalacia in her knees, and ulcerative colitis (which had been diagnosed more than six years earlier and had achieved a drug-induced remission on her current daily balsalazide disodium). She had no history of sores in her mouth, Raynaud’s phenomenon, or lupus erythematosus. Three months earlier, she had been diagnosed with stage II estrogen receptor (ER)-positive, progesterone receptor (PR)-positive, human epidermal growth factor receptor 2 (HER2)-negative T2N1M0 invasive ductal carcinoma of her left breast. Her breast cancer was initially managed with a left breast lumpectomy and sentinel lymph node biopsy. Systemic chemotherapy with Taxotere (Sanofi, Paris, France) and cyclophosphamide was started; however, she experienced a hypersensitivity reaction within 90 seconds after starting the first infusion of Taxotere.

**Figure 1:**
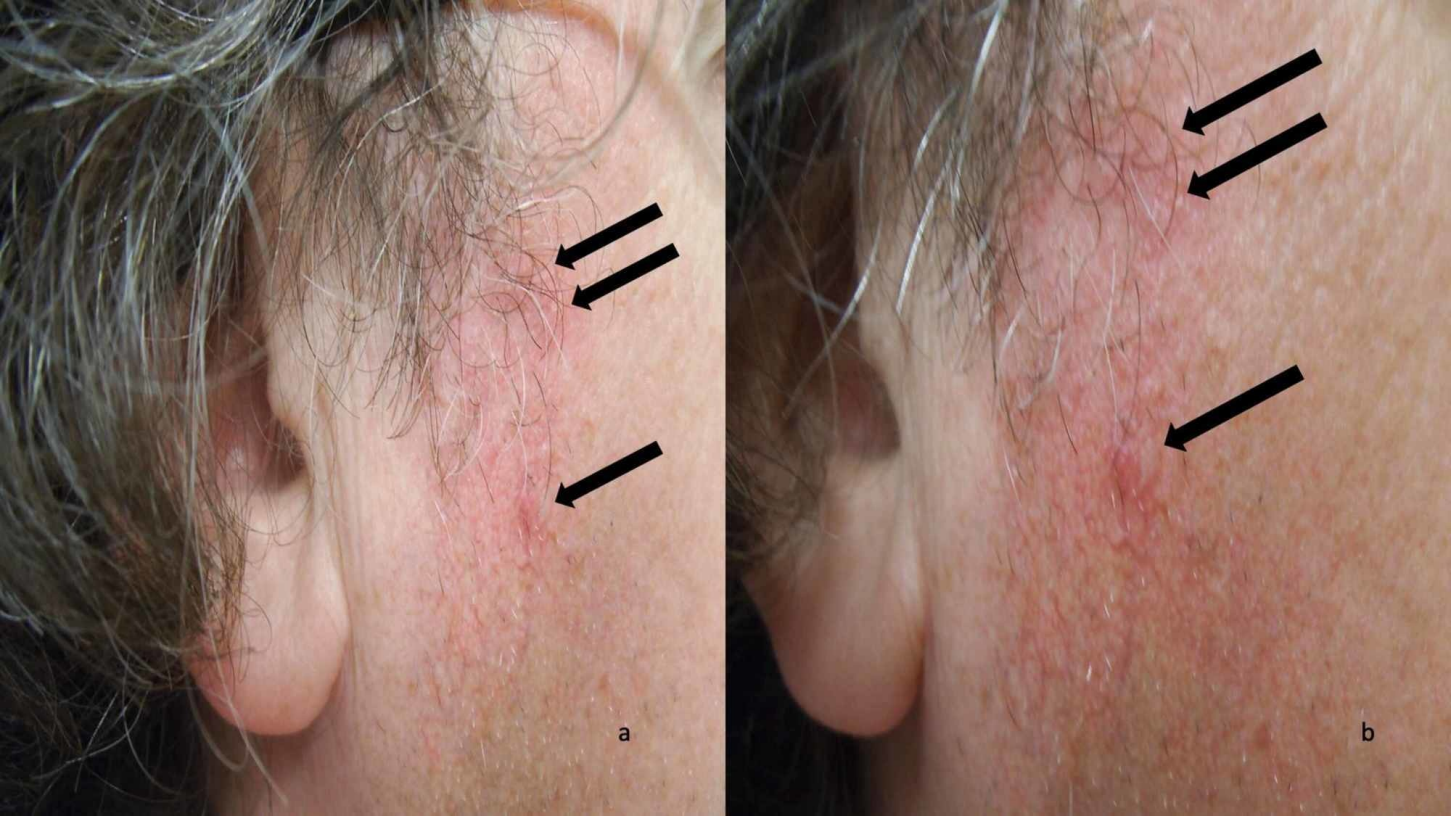
Facial lesions caused by systemic 5-fluorouracil-induced discoid lupus erythematosus Distant (a) and closer (b) views of erythematous papule (lower black arrow) and plaques (upper black arrows) of discoid lupus erythematosus lesions and surrounding telangiectasias on the right preauricular area of the face that appeared three weeks after beginning chemotherapy with 5-fluorouracil

Her chemotherapy was changed to monthly cycles of cyclophosphamide (orally for the first 14 days), and intravenous methotrexate and 5FU on days one and eight of each cycle. Her facial lesions appeared 21 days after her first infusion (and 14 days after her second infusion). They had continued to increase in number and location during the following week. Cutaneous examination showed erythematous papules and scaly patches on her forehead, nose, and chin; similar lesions were also present bilaterally on her cheeks and preauricular areas (Figure 1). In addition, there were telangiectasias on her preauricular areas. There were no other skin lesions, oral ulcerations, or alopecia.

Laboratory evaluation revealed a high positive antinuclear antibody (ANA, with a titer of greater than 1:640; negative, less than 1:40) in a homogenous pattern and a positive anti-double-stranded deoxyribonucleic acid antibody [dsDNA, with an elevated result of 43 international units (IU); negative, 0-24 IU]. Additional studies were negative or within normal limits: complete blood cell count with differential and platelets, serum chemistries, C-reactive protein, erythrocyte sedimentation rate, anti-Ro/Sjogren’s syndrome A (anti-Ro/SSA) antibody, anti-La/Sjogren’s syndrome B (anti-La/SSB) antibody, anti-ribonuclear protein (RNP) antibody, serum complements (C3 and C4), and urinalysis.

Correlation of the history, clinical presentation, and laboratory studies established a diagnosis of systemic 5FU-induced DLE. She was initially treated with desonide 0.05% cream twice daily. Her facial lesion improved with the topical treatment; however, she decided to discontinue using the topical corticosteroid cream after one week since she was concerned about the medication possibly causing skin atrophy. Her oncologist suggested changing her chemotherapy to doxorubicin and cyclophosphamide. However, the patient declined this recommendation and decided to maintain her current chemotherapy regime.

She received her chemotherapy cycles every month; intravenous dexamethasone was also given prior to each infusion of her antineoplastic drugs. The erythematous papules and plaques flared approximately a week after the second infusion during each of her next five chemotherapy cycles. The delay of the facial lesions after treatment was attributed to the temporary prevention of new DLE lesions and the flaring of existing lesions by the systemic corticosteroid. She was seen at her follow-up five weeks after her final cycle of chemotherapy. Her facial DLE lesions were still present; however, they were starting to improve. Laboratory studies demonstrated that her ANA was unchanged (at a titer of greater than 1:640), but her dsDNA was now negative.

Cutaneous examination of her face was performed at her next follow-up visit three months later (more than four months after completing chemotherapy). The DLE lesions on her face had completely cleared. Her ANA had also significantly decreased to a titer of 1:80. Whole-breast radiotherapy had been started approximately one month after finishing chemotherapy. After completion of the radiation therapy, she was started on daily anastrozole. At her four-year follow-up visit, she was found still receiving daily anastrozole; she has had no recurrence of either her breast cancer or 5FU-induced DLE facial lesions.

## Discussion

5FU can be used topically to treat actinic keratoses and superficial basal cell carcinomas and systemically to treat visceral malignancies. Cutaneous side effects following topical use of 5FU may include contact dermatitis (such as allergic or irritant), dermatitis or seborrheic dermatitis (localized or distant from the site of application), erosions, eye-related symptoms (such as conjunctivitis and corneal irritation), a flare of rosacea or seborrheic dermatitis, herpes simplex virus reactivation, nail changes (such as melanonychia and onycholysis), photosensitivity, secondary bacterial infections, telangiectasia formation, and, albeit rarely, neutropenia and other systemic reactions [5-7]. Adverse skin events to systemic 5FU include acral erythema (also referred to as hand-foot syndrome or palmar-plantar erythrodysesthesia), actinic keratosis inflammation, alopecia, hyperpigmentation (of the nails and serpentine supravenous overlying superficial veins), photo-recall reactions (on sun-exposed areas), photosensitivity, and seborrheic dermatitis exacerbation [4,6,8].

Systemic 5FU or its prodrugs (capecitabine, tegafur, and UFT) can result in drug-induced lupus erythematosus. Fluorouracil agent drug-induced lupus erythematosus can present as either SCLE (from capecitabine, systemic 5FU, or UFT) or DLE (from capecitabine, systemic 5FU, tegafur, or UFT) [9-13]. In addition, one study has reported drug-induced lupus erythematosus that presented as SLE in a 68-year-old woman who was receiving capecitabine and bevacizumab for metastatic colon adenocarcinoma [14]. Also, in patients with a prior history of either SLE or DLE, systemic 5FU can cause their lupus to flare [4,15].

Patients with DLE induced by systemic 5FU or its prodrugs have previously been reported as having DLE-like eruptions or DLE-like lesions [12,13]. In this manuscript, the cutaneous manifestations described in the reported patient and those in previously published cases are referred to as DLE lesions. However, some of the patients who were previously reported as having developed DLE-like lesions after receiving UFT were subsequently determined to have drug-induced SCLE since they all had anti-Ro/SSA antibodies; therefore, their characteristics are not included in the clinical and laboratory features of the individuals discussed in this paper [11,16].

To the best of my knowledge, DLE induced by systemic 5FU and its prodrugs has been described in 19 oncology patients (including the patient discussed in this report). There have been nine men and 10 women. The mean onset age of the individuals was 59 years (Table 1) [12,13].

**Table 1:** Clinical characteristics of 19 oncology patients with discoid lupus erythematosus lesions induced by systemic 5-fluorouracil or its prodrugs A: age (in years); C: case; Cap: capecitabine; Cau: Caucasian; Ch: chin; Ck: cheek; Cks: cheeks; CR: current report; DLE: discoid lupus erythematosus; ER: estrogen receptor; Fa: face; Fh: forehead; 5FU: 5-fluorouracil; HER2: human epidermal growth factor receptor 2; Ita: Italian; IVD: intravenous dexamethasone; Jap: Japanese; No: nose; Pa: preauricular area; PR: progesterone receptor; R: race; Rt: right; Sp: stop; TCs: topical corticosteroid; UFT: uracil/tegafur; W: woman ^a^Cases 4-19: nine men and seven women with “DLE-like eruption type” of skin eruptions induced by fluorouracil agents; the data were summarized by Yoshimasu et al. [13]. Their average age (mean) was 59 years. The causative drug was either tegafur (10 cases), UFT (five cases), or 5FU (one case); the mean dose of the fluorouracil agent was 139 grams. The patients' cancers were not stated. The clinical appearance of the DLE-like eruption was not described; however, the favored sites of the lesions were sun-exposed areas. The mean duration time before the onset of DLE after starting the fluorouracil agent was 261 days. The mean regression time of the DLE eruption after drug discontinuation was 34 days (range: 7-60 days) ^b^The onset duration is the number of weeks from the patient starting the drug to the appearance of the DLE lesions ^c^The resolution duration is the number of weeks from the patient stopping the drug to the complete clearing of the DLE lesions ^d^The woman had a moderately differentiated adenocarcinoma of the rectum (pT3N1M0) ^e^Initial episode ^f^Rechallenged with capecitabine ^g^UFT total dose was 42 grams ^h^DLE-like eruption did not regress ^i^The woman had stage II ER-positive, PR-positive, HER2-negative T2N1M0 invasive ductal carcinoma of her left breast ^j^DLE lesions improved; however, medication was stopped ^k^Premedicated prior to each 5-FU infusion

C^a^	A/R/sex	Drug	Cancer	Clinical appearance	Site	Onset^b^	Treatment	Resolution^c^	Reference
1	58/Ita/W	Cap	Rectal^d^	Erythematous rash^e^; Erythematous rash^f^	Cks^e^; Fa and scalp^f^	10^e^; 4^f^	Sp Cap^e^; Sp Cap^f^	8^e^; 4^f^	[12]
2	64/Jap/W	UFT^g^	Lung	Round erythema	Rt Ck	20	TCs^h^, Sp UFT	8	[13]
3	65/Cau/W	5FU	Breast^i^	Red papules and scaly plaques	Fh, No, Ch, Pa, and Cks	3	TCs^j^, IVD^k^, Sp 5FU	17	CR

Tegafur was the most common medication associated with inducing DLE (10 patients). UFT was responsible for drug-induced DLE in six individuals. Less commonly, systemic 5FU (two patients) and capecitabine (one patient) also induced DLE lesions (Table 1).

Clinically, the DLE lesions are found on sun-exposed areas such as the scalp and/or face. They may present as round erythema or an erythematous rash [12,13]; one woman’s lesion was like a butterfly rash on her face [12]. The woman in this report had red papules and scaly plaques on her face including not only her nose and cheeks but also her forehead, chin, and preauricular areas.

The mean onset of the DLE lesions after initiating fluorouracil agent chemotherapy in the 19 patients was 232 days; however, 84% (16 of 19) of these patients had been treated with either tegafur or UFT. Shorter onset periods were observed in the three women for whom specific information was available; in these individuals, the onset for the appearance of DLE lesions was either 21 days (after starting systemic 5FU) or 70 days (after starting capecitabine), or 140 days (after starting UFT) (Table 1). It is possible that the conversion of the prodrug to 5FU may account for the varying delay in the onset between starting treatment with the fluorouracil agent and the appearance of the DLE lesions. Topical corticosteroids were used in the management of two of the women. A low-potency corticosteroid (desonide 0.05%) cream resulted in prompt improvement in the reported patient; however, she was concerned about the potential side effects of the medication and stopped using it. Another woman was treated with a topical corticosteroid therapy for two months; however, she continued to receive daily UFT and the round erythema on her right cheek did not regress [12].

The regression time of the DLE lesions after stopping the fluorouracil drug ranged from seven to 60 days (with a mean of 36 days) in 95% (18 of 19) of the patients (Table 1). Complete clearing of the facial DLE lesions was recorded at a follow-up visit 119 days after stopping systemic 5FU in the reported patient. However, her facial lesions had resolved between 4-17 weeks after her chemotherapy treatment had been discontinued. The woman with capecitabine-induced DLE was restarted on the agent two months after it had been discontinued and her facial DLE lesions had improved. Initially, she had developed an erythematous rash of drug-induced DLE on her cheeks 10 weeks after starting capecitabine. After beginning the rechallenge with capecitabine, the DLE lesions appeared on her face and scalp within four weeks; the drug was stopped and the DLE lesions subsequently resolved within the next four weeks [12].

Laboratory features of the oncology patients with fluorouracil agent-induced DLE are summarized in Table 2 [12,13]. A biopsy of the skin lesion was performed, and it resulted in pathologic changes of DLE in two of the women [12,13]. A skin biopsy was also recommended for the patient in this report; however, she declined to have the procedure.

**Table 2:** Laboratory features of 19 oncology patients with discoid lupus erythematosus lesions induced by systemic 5-fluorouracil or its prodrugs ANA: antinuclear antibody (less than 1:40 is negative); Bx: biopsy of the skin; C: case; CC: complement component levels; CR: current report; D: declined; DLE: discoid lupus erythematosus; dsDNA: double-stranded deoxyribonucleic acid antibody (less than 25 IU is negative); ENA: extractible nuclear antigen; 5FU: 5-fluorouracil; La/SSB: Sjogren’s syndrome B; IU: international units; ND: not done; Oth: other laboratory studies; RF: rheumatoid factor; RNP: ribonuclear protein; Ro/SSA: Sjogren’s syndrome A; UFT: uracil/tegafur; UA: urinalysis ^a^Cases 4-19: 16 patients with “DLE-like eruption type” of skin eruptions induced by fluorouracil agents; the data were summarized by Yoshimasu et al. [13]. The ANA was positive for 64% (nine of 14) of the patients ^b^Performed when women presented for evaluation of DLE lesions ^c^Number of weeks after the fluorouracil agent was discontinued ^d^58-year-old woman; received capecitabine ^e^Right malar cheek biopsy showed atrophy of the epidermis, slight liquefaction of the basal cell layer, and patchy lymphocytic infiltration in the perivascular and perifollicular regions. Direct immunofluorescence examination was negative ^f^Waaler-Rose titer was slightly elevated (1:40). Normal or negative results: anti-ENA antibodies, RF, serum protein electrophoresis, UA, and white blood cell count ^g^64-year-old woman; received UFT ^h^Cheek biopsies showed atrophic epithelium, liquefaction of the basal cell layer, and perivascular and perifollicular lymphocytic infiltration in the dermis ^i^RF was slightly elevated (76 IU per mm) ^j^65-year-old woman; received 5FU ^k^A skin biopsy was recommended; however, the patient declined ^l^ANA pattern was homogenous ^m^Normal or negative results: C-reactive protein, complete blood cell count with differential and platelets, erythrocyte sedimentation rate, anti-RNP antibody, serum chemistries, and UA

C^a^	Bx	Initial ANA^b^	Initial ds DNA^b^	Ro/SSA	La/SSB	CC	Oth	Follow-up time^c^	Follow-up ANA	FUp ds DNA	Reference
1^d^	+^e^	1:160	-	-	-	Nl	+^f^	ND	ND	ND	[12]
2^g^	+^h^	1:80	-	-	-	Nl	+^i^	8	1:20	ND	[13]
3^j^	D^k^	>1:640^l^	43 IU	-	-	Nl	+^m^	5; 17	>1:640^l^; 1:80^l^	22 IU; ND	CR

The ANA was evaluated in 17 of the 19 patients with fluorouracil agent-induced DLE (Table 2). A positive ANA was observed in 71% (12 of 17) of the individuals. Specific ANA titers were available for three women: greater than 1:640 with a homogenous pattern (in the systemic 5FU recipient), 1:160 (in the capecitabine recipient), and 1:80 (in the UFT recipient). Additional serologic testing was provided for three of the women (Table 2). All of the women had normal complement component levels. None of the patients had antibodies to Ro/SSA or La/SSB. The reported patient had a positive antibody titer to dsDNA; however, this antibody titer became negative within five weeks after stopping the systemic 5FU.

Follow-up ANA titers were performed for two of the women. The ANA titer decreased from 1:80 to 1:20 eight weeks after stopping UFT in the woman with lung cancer [13]. The ANA titer remained at greater than 1:640 five weeks after stopping systemic 5FU in the reported patient with breast cancer; however, the titer significantly decreased to 1:80 on a subsequent evaluation 17 weeks after discontinuing the medication.

The pathogenesis for DLE lesions after initiating treatment with systemic 5FU and its prodrugs remains to be determined; investigations into the mechanism for the development of drug-induced cutaneous lupus erythematosus continue to be pursued [17]. None of the patients, for whom information was available, had antibodies to Ro/SSA or La/SSB. Also, the observed clinical improvement of the facial DLE lesions directly correlated with the decreased antibody titers to ANA (Tables 1, 2).

## Conclusions

DLE induced by systemic 5FU and its prodrugs has been described in 19 oncology patients in the literature so far (including the patient discussed in this report): tegafur (10 patients), UFT (six patients), systemic 5FU (two patients), and capecitabine (one patient). The mean duration before the appearance of the erythematous patches, papules, and/or scaly plaques on sun-exposed areas was nearly eight months after beginning the fluorouracil agent; however, they appeared within three weeks after starting systemic 5FU in one of the women. The DLE lesions resolved within two months (mean: 36 days) after stopping the drug in 95% of the patients. Laboratory studies showed that antibodies to Ro/SSA and La/SSB were negative in 100% (three of three) of the patients. The elevated ANA titer was found to be decreased in all of the patients who were evaluated after the discontinuation of the drug. The definitive etiology for fluorouracil agent drug-induced DLE remains to be established.
